# Contested professional role boundaries in health care: a systematic review of the literature

**DOI:** 10.1186/s13047-015-0061-1

**Published:** 2015-02-05

**Authors:** Olivia King, Susan A Nancarrow, Alan M Borthwick, Sandra Grace

**Affiliations:** School of Health and Human Sciences, Southern Cross University, Lismore, NSW 2480 Gold Coast, Australia; Centre for Innovation and Leadership in Health Sciences, Faculty of Health Sciences, University of Southampton, Southampton, SO17 1BJ England

**Keywords:** Contested boundaries, Professional boundaries, Inter-professional boundaries

## Abstract

**Background:**

Across the Western world, demographic changes have led to healthcare policy trends in the direction of role flexibility, challenging established role boundaries and professional hierarchies. Population ageing is known to be associated with a rise in prevalence of chronic illnesses which, coupled with a reducing workforce, now places much greater demands on healthcare provision. Role flexibility within the health professions has been identified as one of the key innovative practice developments which may mitigate the effects of these demographic changes and help to ensure a sustainable health provision into the future. However, it is clear that policy drives to encourage and enable greater role flexibility among the health professions may also lead to professional resistance and inter-professional role boundary disputes. In the foot and ankle arena, this has been evident in areas such as podiatric surgery, podiatrist prescribing and extended practice in diabetes care, but it is far from unique to podiatry.

**Methods:**

A systematic review of the literature identifying examples of disputed role boundaries in health professions was undertaken, utilising the STARLITE framework and adopting a focus on the specific characteristics and outcomes of boundary disputes. Synthesis of the data was undertaken via template analysis, employing a thematic organisation and structure.

**Results:**

The review highlights the range of role boundary disputes across the health professions, and a commonality of events preceding each dispute. It was notable that relatively few disputes were resolved through recourse to legal or regulatory mandates.

**Conclusions:**

Whilst there are a number of different strategies underpinning boundary disputes, some common characteristics can be identified and related to existing theory. Importantly, horizontal substitution invokes more overt role boundary disputes than other forms, with less resolution, and with clear implications for professions working within the foot and ankle arena.

## Background

Health care services in the UK, Australia and elsewhere across the Western world are under significant pressure to provide innovative, effective and timely services to an ever growing and ageing population [1,2]. Trends in demographic data suggest that an unprecedented rise in chronic and complex health care conditions will continue to emerge, alongside advancing technology, economic uncertainty and financial restraint, all of which constitute a major challenge for healthcare policy [3,4].

For foot and ankle specialists in Australia and the UK, the prevalence of diabetes is increasing more rapidly than any other chronic health condition, and workforce requirements already exceed current levels of supply [5]. In Australia, there are 1.1 million people currently living with diabetes, with approximately 100,000 new cases diagnosed each year [6]. Diabetes-related complications impact not only the individual, their quality of life and life expectancy, but also place significant burden on an already stretched health care system [6].

In light of these challenges, health policy responses support professional role flexibility and innovation in health care practice, in order to meet population health needs [7,8]. Increasingly there has been a strategic move towards interprofessional practice, with an increased likelihood of role blurring. Interprofessional practice describes a team-based approach to health service provision, hinging upon a willingness to abandon traditional role boundaries and relinquish claims of exclusivity to health care practices and knowledge [9]. This blurring of boundaries in the health care workforce may see different professions taking on practices previously “owned” by others [7] thus shifting the focus from the professions themselves, to better meet the health needs of the service-user [8]. However, uncertainty around role delineations and service funding constraints militate against unproblematic and seamless transitions [10]. Inter-professional disputes involving allied health professions concerned with foot and ankle work, for example, have been well documented but nevertheless remain rare [10-13]. A broader exploration of the strategies, forms and outcomes of healthcare boundary disputes across the healthcare professions is likely to yield deeper insights into the complexities and intricacies of role boundary disputes, and thus better inform future policy decisions on feasible solutions – solutions which may equally apply across the health sector [14].

Social theory also provides insights into the way in which role boundary disputes operate [15-18]. Occupational closure is a widely recognised means of attempting to secure exclusivity, most powerfully via legal and regulatory mechanisms, such as certification and credentialing processes, leading to a “market shelter” [19]. However, strategies to ensure the exclusion of competitors is necessarily threatened by the advent of workforce flexibility models intent on imposing role re-design [20,21]. Most commonly, healthcare role boundary disputes emerge between medicine and other non-medical professions seeking to acquire roles previously exclusive to medicine [15,22-24].

However, relatively few papers address boundary disputes between professions considered to be of equal status [25]. It was therefore important to view the literature across all the healthcare professions, and not confirm the search to studies of disputed medical boundaries alone. The literature review seeks to explore reported boundary disputes, how these came about, what these contests or disputes looked like and what, if any, outcomes resulted from these inter-professional role boundary contests.

## Methods

### Literature search

Only peer reviewed research articles were considered for inclusion in the literature review. A STARLITE framework was used to guide the search [26]. See Table 1 for STARLITE framework detailing search strategy including terms, databases, inclusions and exclusions.

**Table 1:** **STARLITE framework**

**Sampling strategy**	**Purposive**
Type of literature	Qualitative and quantitative research
Approaches	Subject search, citation search, internet search
Range of years	No start date to March 2014
Limits	English, human
Inclusions and exclusions	**Included:** Health or health care and: contested role boundaries, disputed role boundaries, role boundary negotiation, interprofessional role boundaries, professional role boundaries
	**Excluded:** Discussion pieces; developing countries; intra-professional boundary disputes; papers referring to, but not focusing on interprofessional boundary disputes; complementary and alternative therapies *only*; interprofessional education; collaboration; multidisciplinary care
Terms used	Contested boundaries AND health care
	Dispute* boundary$ AND health care
	Professional boundary$ AND health care
	Interprofessional boundary$ AND in health
Electronic resources	CINAHL, Medline, PubMed, Expanded Academic ASAP

The symbols * and $ constitute literature search strategy Boolean connectors. * is a wildcard symbol replacing one letter in a search term, enhancing a database search. $ is a truncation symbol which allows retrieval of words in both plural and singular.

### Synthesis and analysis

In synthesising and analysing the findings from the seven papers, a framework analysis akin to template analysis, described by Brooks and King [27] is used. Template analysis is a technique which employs thematic organisation and analysis of data. The data being analysed can be any kind of textual data however interview transcripts are the most common form of data for which this approach is used. The key to this form of analysis is the use of a priori template, or pre-determined code set, to extract, organise and analyse qualitative data.

The pre-determined template used to code and analyse data in this literature review is the framework described by Nancarrow and Borthwick (2005) [8] which relates to the dynamic health care workforce. The authors describe the directions in which workforce boundaries can shift. These directions of role boundary shifts serve as a priori themes in which the papers will be coded.

Diversification is the identification of a new approach to practice that has previously been claimed and “owned” by another profession. It may mean the creation of a new task or it may be performing an existing task with a new method, resulting in professional role expansion. Specialisation is defined by Nancarrow and Borthwick (2005) [8] as “the adoption of an increasing level of expertise in a specific disciplinary area that is adopted by a select group of the profession and legitimised through the use of a specific title, membership to a closed sub-group of the profession, and generally involves specific training”. Specialisation is more successful at post-registration level.

Boundary expansion can be also achieved by role substitution. Substitution see a profession taking on work previously undertaken by another profession and can occur vertically or horizontally. Vertical role substitution refers to the delegation or acquisition of tasks across professional boundaries where one profession is considered to be superior on the hierarchy. The degree to which vertical substitution expands role boundaries is highly dependent on the profession considered to be superior. Boundary expansion can also occur by horizontal substitution, which occurs when a profession considered to be have a similar level of expertise and training as another one, takes on part of their traditional role. For this form of role expansion to be successful roles must be quite flexible to enable other professions to adopt them. Horizontal substitution is thought to be occurring more frequently with the rise in interprofessional education and practice [8].

## Results

**Figure 1:**
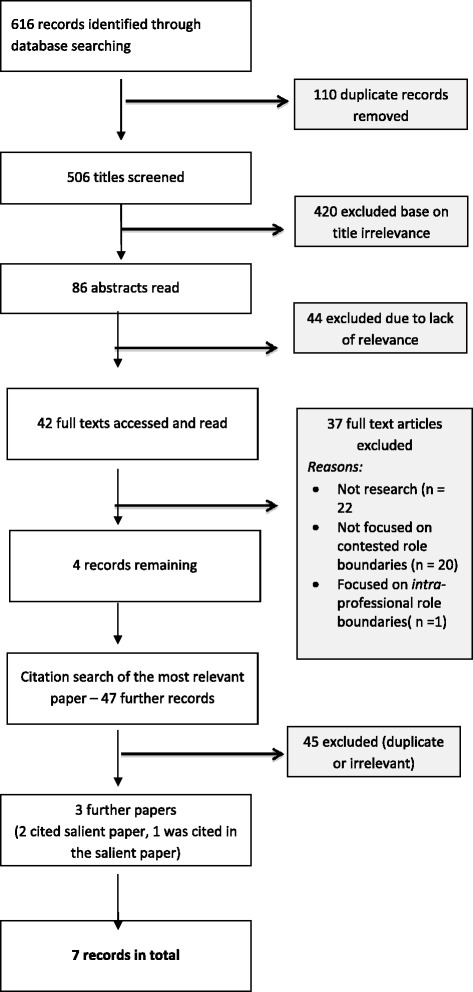
**PRISMA diagram - pictorial summary showing the location of papers at each stage of the literature search process.**

Initial database searching using the terms in the STARLITE framework, yielded 616 results. See Figure 1 for a pictorial summary showing the location of papers at each stage of the literature search process.

Studies which focused on contested role boundaries; including the drivers for contestation, evidence of boundary disputes and the means by which professions defended their occupational territory were included. Papers referring to boundary disputes but not focused on them and those looking at intra-professional boundary disputes, were excluded. See STARLITE framework (Table 1) for details of inclusions and exclusions. With only four papers remaining, a citation search of the paper which seemed most relevant [25] was undertaken. This turned up a further three papers; two citing the relevant paper, one cited by its authors. There were seven papers in total included in the literature review and were published between 2001 and 2012.

### Quality indicators

The seven selected papers were assessed for quality using the critical appraisal skills programme (CASP) qualitative research checklist [28]. See Table 2.

**Table 2:** **Quality indicators**

**Quality indicator**	**Yes**	**Can’t tell**	**No**
Was there a clear statement of the aims of the research?	7	0	0
Is a qualitative methodology appropriate?	7	0	0
Was the research design appropriate to address the aims of the research?	7	0	0
Was the recruitment strategy appropriate to the aims of the research?	7	0	0
Was the data collected in a way that addressed the research issue?	6	1	0
Has the relationship between the researcher and the participants been adequately considered?	1	6	0
Have ethical issues been taken into consideration?	7	0	0
Was the data analysis sufficiently rigorous?	6	1	0
Is there a clear statement of findings?	7	0	0
Is the research valuable?	7	0	0

All of the included studies used qualitative methodology, which is well-suited to the topic. Table 3 provides an overview of the main characteristics of each of the included papers.

**Table 3:** **Main characteristics of included studies**

**Authors**	**Overview**	**Country**	**Study type**	**Setting**	**Professions involved**
Bach et al. 2012 [4]	Looks at boundary work undertaken by registered nurses and health care assistants (HCAs) working in two National Health Service (NHS) Trusts	UK	Qualitative research using data from 60 semi-structured interviews	Two different hospitals	34 HCAs and 26 Registered Nurses, including senior nurses and sisters
Martin et al. 2009 [29]	Looks at micro-level professional jurisdiction negotiations between GPs with special interest (GPSI) in genetics and clinical geneticists	UK	Qualitative research using data from 34 in-depth interviews with GPSIs, clinical geneticists and other key stakeholders	Four different genetics clinics included in a pilot program	GPSI, clinical geneticists, managers and other staff working in the field of clinical genetics at the pilot sites
McIntyre et al. 2012 [30]	Analysis of the perspectives of the prominent service providers in maternity care on proposed service reforms	Australia	Critical discourse analysis; data obtained by 24 selected submissions to the maternity services review in 2008	Not specified	Professional associations including obstetrics, midwifery, rural doctors, GPs, academic institutions, women’s health networks, hospitals and the Australian Medical Association
Norris 2001 [31]	Looks at micro-level boundary work undertaken by a large range of orthodox and alternative practitioners treating musculo-skeletal pathologies.	New Zealand	Qualitative research drawing data from semi-structured interviews with 83 treatment providers and 13 professional associations	Interviews took place mainly in the workplaces of the interview participants	Seven medical specialists, 17 GPs, 17 physiotherapists, eight chiropractors, osteopaths and massage therapists, four acupuncturists, two Alexander technique practitioners, podiatrists, psychologists and beauty therapists (massage)
Salhani and Coulter 2009 [32]	Explores the micro-political struggles within an interprofessional mental health team working in a mood disorder unit. Focuses on politics and power, with an emphasis on nursing’s professional project.	Canada	Qualitative research using an ethnographic approach. Data obtained by intensive observation of the interprofessional team while at work, formal interviews with unit staff and review of relevant documents	A mood disorder unit in a metro psychiatric hospital	Interviews were conducted with seven psychiatric nurse assistants, six psychiatric nurses, two psychiatrists, psychiatric residents and social workers, one medical resident, head nurse, psychologist, research coordinator, occupational therapist, physiotherapist, pharmacist, chaplain and ward clerk and senior administrators.
Sanders and Harrison 2008 [20]	Looks at the claims of professional legitimacy in heart failure care made by three types of medical specialities and specialist heart failure nurses	England	Qualitative research looking at the content of discourses made by four professions. Data was obtained via semi-structured interviews	Participants’ workplaces (hospital or general practice)	Eight cardiologists, eight geriatricians, seven GPs and ten specialist heart failure nurses
Timmons and Tanner	Explores the case of theatre nurses and operating department practitioners (OPDs) and their disputed occupational boundaries	England	Qualitative research using observation and follow up semi-structured interviews	Sample drawn from five theatre departments across four NHS trusts	Seventeen theatre nurses and three ODPs

The majority of papers focused on the micro-level strategies employed by individual professionals at a local level in order to protect their role boundaries. Macro-level strategies are typically implemented by the professional associations; however the objective - to construct and defend their occupational boundaries - is the same as micro-level. A comparison of the main factors related to the role boundary disputes within the seven papers is presented in Table 4.

**Table 4:** **Features of the role boundary disputes**

	**Feature**	**Number of studies**
Existing or new professions and boundaries	New occupation or sub-speciality encroaching on another’s established boundary	4
	Existing professional group encroaching on another’s established role boundary	1
	Long-standing role boundary contest	2
Overt or subtle contest	Overt boundary contest	4
	Subtle boundary contest	2
	Unclear	1
Professional hierarchy	Hierarchal component	4
	No hierarchal component	2
	Mixed	1
Strategy level being explored in review	Macro-level	1
	Micro-level	5
	Both	1
Initial driver for change in professional role boundary/ies	Government/modernization agenda for health care	2
	Chronic illness trajectory	1
	Shortage of health care professionals	1
	Consumer/community driven	1
	Not specified	2

### Template analysis

Nancarrow and Borthwick’s [8] framework describing the means by which professions can expand their scopes of practice, often into the domain of other existing professions, is used to categorise how each of the boundary disputes came about. See Table 5.

**Table 5:** **Template analysis**

**Strategy to expand role boundaries**	**Authors year**	**Professions involved**	**Overt or subtle dispute**	**Boundary work strategies observed**	**Outcome/s of dispute observed in study**
Diversification	Norris 2001 [31]	A range of practitioners working with musculo-skeletal pathologies including physiotherapists, orthopaedic surgeons, chiropractors, massage therapists and others	Subtle	Occupations made claims of their ability to provide superior musculo-skeletal treatment based on concepts including: others being limited (because they lack something), their approach being holistic (where others are too focused) and prevention as part of their practice	Although professions somewhat succeeded in distinguishing themselves from others, and in some cases pointing out their advantages, it does not appear as though any professions are effectively limiting the practice of others
Specialisation	Martin Currie and Finn 2009 [29]	GPs with special interest in genetics (GPSIs) and clinical geneticists	Overt	GPSIs were eager to extend their skills vertically and practice clinically however geneticists were protective of their professional boundaries. Geneticists argued the indeterminacy of their knowledge, lengthy training and ongoing interaction with a team of experts as their unmitigated advantage over GPSI. GPSIs cited their autonomy as a GP as a strength	The highly specialised status of the geneticists was effectively used to limit the ability of GPSIs to practice in a clinical capacity in genetics. Geneticists successfully limited GPSIs from encroaching on their role, in this particular case
	Salhani and Coulter 2009 [32]	Psychiatric nurses, psychiatrists, occupational therapist, social worker and other allied health professionals and unit managers	Overt	Significant gains of power were made by psychiatric nurses in a setting which traditionally saw medicine (psychiatrists) in a more powerful position. Psychiatric nurses exercised a number of tactics to gain allies in other allied health professions and managerial support, which enabled them to establish their treatment model which contradicted the psychiatric model	Psychiatric nurses were able to not only expand their scope of practice by way of specialisation, they were able to exert their influence and power to achieve a level of autonomy from psychiatry and prevent encroachment from other non-medical professions
	Sanders and Harrison 2008 [20]	Geriatricians, Cardiologists, GPs and heart failure nurses	Subtle	The authors identified four prominent discourses that were used by the heart failure care professional groups, to establish their professional legitimacy and emphasise their advantage over the other professions. These were: expertise, competence, organisational efficiency and patient-centredness	Overt boundary disputes were not evident. Although reluctance of the medical professions to inter-refer may indirectly limit the involvement of certain professions, the role boundaries of one profession are unaffected by another
Vertical Substitution	McIntyre et al. 2012 [30]	Medicine (including specialist obstetricians, general practitioners (GPs) and rural doctors), and midwives (nurses)	Overt	Vertical substitution enabled obstetrics to dominate maternity services. Midwives and their related professional associations birth as a normal, non-medical occurrence. Obstetrics and their professional associations, emphasised the risks associated with childbirth and the importance of a medical professional adopting a senior role in each case	Authors concluded that the historically elite position of obstetrics in maternity care is being challenged by not only midwifery, but also by consumers, maternity service managers and even some medical professions
Horizontal substitution	Bach, Kessler and Heron 2012 [4]	Registered nurses and health care assistants (HCAs)	Overt	The boundary preservation work of the registered nurses focused on attempts to distinguish themselves from the HCAs and assuming an authoritarian role. Alternatively HCAs emphasised their similarity to nurses and their team-based approach to patient care. HCAs were eager to blur the lines between their role and nursing, where nurses were keen to reinforce the divide	Although HCAs are treated as an inferior, marginalized group, nurses appeared unable to prevent them from undertaking traditional nursing work, especially direct patient care activities
	Timmons and Tanner 2004 [25]	Theatre Nurse and Operating Department Practitioners (ODPs)	Overt	Both theatre nurses and ODPs used atrocity stories to illustrate the advantage of their profession over the other. Atrocity stories were categorised into themes: the role of technology; doctor-support versus caring for patients; being patient centred; and the status of Operating Department Practice as a “proper profession”	Theatre nurses did not appear to be able to prevent encroachment on, or extend their own role boundaries

In the only case of diversification as the method used by professions to expand and define their scopes of practice, efforts to distinguish their profession from others did not appear to result in other professions being limited in their practice. There were three cases in this review where specialisation was used by the professions involved to expand their scopes of practice. In two of these cases their efforts to distinguish themselves from other professional groups practicing in the same field did not result in limitation of their practice, however in one case, it did. In this particular case, involving clinical geneticists and GPs with special interest (GPSI) in genetics, there was a hierarchal component involved which was effectively used by the clinical geneticists to limit the practice of GPSI.

There was one case illustrating vertical substitution, implicating professions directly involved in maternity care. This is actually an example of vertical substitution in reverse. Maternity care was once undertaken by lay women however medicine commandeered the practice and medicalised it to the point where it became the medical speciality known as obstetrics [8]. In McIntyre et al. [30] study, they found that some progress had been made on the part of midwifery to regain some of the power assumed by obstetrics in maternity care services.

There were two examples of horizontal substitution as the means by which professional groups expanded or defined their practice. Both of these examples were new professions whose core work was considered part of nursing’s traditional work domain. Both of the relatively new professions sought to highlight their similarities to, and in one case, their advantage over nursing in their particular work domain. While nurses sought to distinguish themselves and cite their superior capabilities in both cases, this did not appear to hamper the ability of the newer professions in encroaching on nursing’s traditional occupational territory.

## Discussion

Specific role boundary changes in allied health professions such as podiatry, have been captured in papers deploying social theory and these give some flavour of the type and form of boundary shifts and disputes that have emerged to date [10,33-35]. However, a broader sweep of literature across the health sector gives a clearer picture of the complex relationships giving rise to boundary disputes which may impact on other professions concerned with foot and ankle work.

It is clear that disputes between the professions take various forms, some more overt than others. However, the events preceding role boundary contests appear to be similar and revealing. In each case selected there was either a shortage of qualified staff to undertake a particular healthcare role, or an underpinning governmental modernization agenda driving role change to alternative providers, the latter often involving a community or consumer drive, prompting the genesis of a new profession in some cases and the extension of scope of practice in others. Both sought to establish themselves and their role boundaries by way of diversification, specialisation, vertical or horizontal substitution, often into the domain of an existing profession [8]. In each instance, where the profession concerned enjoyed established roles, it sought to protect them when threatened, with the response more vociferous when the boundary was perceived to be more vulnerable.

Some of the selected studies focused on those strategies employed by the encroaching profession, others on those defending boundaries. In each case, the common theme derived from the use of discourse to discredit the competitor profession, either on the basis of their approach to clinical care or their skills or competence. There were several references made to macro-level strategies adopted by some professional associations, where emphasis was placed on highlighting the advantages of one profession over a neighbouring one [31]. However, in all cases the micro-level strategies employed by professionals were at the forefront of the discussion.

In cases where the focus was on resisting encroachment on an existing occupation’s domain, both success and failure were demonstrated. Unlike much of the theory of medical dominance, in which medical specialities would, by virtue of their social and cultural authority, be more successful at protecting boundaries and resisting change, evidence in the studies selected suggest a change, based on aligning demands with policy direction. Salhani and Coulter [32], for example, found in their study of nurses working within a multidisciplinary team in a mood disorder unit, that nurses were successful in securing autonomy from psychiatry, a move which enabled them to overrule psychiatrists intervention, whilst also resisting encroachment from other professions within the unit; a success based upon an overarching policy need for workforce flexibility.

In a study looking at the boundary work of both registered nurses and health care assistants (HCAs) in two NHS Trusts, Bach et al. [4] found that the nurses enthusiastically defended their unique ability to provide hands-on, patient-centred, holistic care. In this study, the nursing profession was portrayed as one seeking to expand their role boundaries into the domain of medicine whilst also preserving their current boundaries, thus preventing usurpation by HCAs – what Witz referred to as ‘dual closure’ [36]. However, the nurses were also keen to shed the “dirty work”, which proved to be a source of frustration for the HCAs, which thwarted their own professionalising ambitions [37].

In each of the five studies involving the nursing profession, references to “patient-centred” and “holistic care” were made, clearly as a form of professional rhetoric designed to support their bid for legitimacy in claiming role exclusivity, or at least primacy. In Timmons and Tanner’s [25] study the overt boundary dispute between theatre nurses and operating department practitioners (ODPs), nurse claims to a broad caring and holistic role gave them a perceived advantage over the ODPs, particularly in relation to gender claims, with nurses often referring to the insensitive masculine approach to patient care exhibited by the ODPs.

Similar to Timmons and Tanner’s [25] study, podiatrists utilise horizontal substitution as a means to expand their scope of practice into the diabetes educator domain. This type of role boundary expansion has been shown to lead to hotly contested role boundary disputes, between two professions of equal standing, yet waged across a field in which one occupies the ‘established’ role and the other the ‘usurper’ role [17].

### Methodological limitations of the literature review

Whilst taking a systematic approach to this literature review confers a degree of objectivity, there is a risk that some relevant literature may have been omitted, such as grey literature, or that not all the relevant literature has been included. Literature which did not constitute research, nor actually focus on a disputed boundary was excluded, meaning that some material on role boundary disputes in health care was not considered. Equally, some databases which were not within the health field may have contained relevant papers shedding light on boundary disputes relevant to healthcare, such as PsycINFO or SocINDEX.

Generalisability of the findings is made difficult by the fact that only seven papers met the inclusion criteria and the use of qualitative methodologies rendered this problematic. With relatively small sample sizes, including some single case studies, application of the analysis adapted to broader health care settings was not considered appropriate. Comparing and contrasting the finding of the papers was made difficult by the fact the various studies used different themes to code and analyse their data.

## Conclusion

This literature review has explored cases of contested role boundaries, demonstrating that there are many different kinds of boundary disputes among the health professions, each with varying emphases and outcomes. The means by which professions attempt or succeed in extending their role boundaries - diversification, specialisation, vertical substitution or horizontal substitution - does not appear to directly influence whether or not a profession is able to extend its role boundaries where these are contested. However it is clear that horizontal substitution appears to invoke more overt role boundary disputes, with little likelihood of a negotiated resolution.

Persistent health professional role boundary disputes may impede role flexibility and are likely to undermine the development of the health care workforce in line with policy objectives aimed at workforce redesign and longer term service sustainability in the face of demographic change. Trends in health policy continue to support professional role boundary flexibility and innovation in service delivery, and the recent exemplar of extensions in non-medical prescribing illustrates the impact of role redesign on health service provision and highlights specific directions in policy implementation, governance frameworks and competency structures to support their implementation. Role boundary disputes may delay, but are unlikely to prevent, full implementation of policy and practice developments.

This literature review supports the widely accepted notion that the health care division of labour is based not on immovable professional boundaries, but on dynamic shifts influenced by forces such as the health policy agenda, and may not always favour the traditionally most powerful professions. It may signal a reduction in professional power and autonomy by some of the professions, each of which is increasingly vulnerable to the vagaries of the healthcare market, and the fiscal restraints imposed on healthcare budgets.
